# The efficacy and safety of niraparib for ovarian cancer: a single-center observational study from China

**DOI:** 10.1186/s13048-021-00803-2

**Published:** 2021-05-17

**Authors:** Jing Ni, Xianzhong Cheng, Qian Zhao, Zhiqin Dai, Xia Xu, Wenwen Guo, Hongyuan Gu, Rui Zhou, Yan Wang, Xiaoxiang Chen

**Affiliations:** 1 Department of Gynecologic Oncology, Jiangsu Cancer Hospital, Jiangsu Institute of Cancer Research, The Affiliated Cancer Hospital of Nanjing Medical University, 42# Baiziting street, Nanjing, Jiangsu 210009 People’s Republic of China; 2 Department of Chemotherapy, Nanjing Medical University Affiliated Cancer Hospital, Jiangsu Cancer Hospital, Jiangsu Institute of Cancer Research, 42# Baiziting street, Nanjing, Jiangsu 210009 People’s Republic of China; 3 Department of Pathology, The Second Affiliated Hospital of Nanjing Medical University, 121# Jiangjiayuan Road, Nanjing, Jiangsu 210011 People’s Republic of China

**Keywords:** Ovarian cancer, Niraparib, Real world, Efficacy, Safety, HRD

## Abstract

**Background:**

Niraparib, a poly (ADP-ribose) polymerase (PARP) inhibitor, is approved for first/second-line maintenance treatment of ovarian cancer patients with complete or partial response to platinum-based chemotherapy, and multi-line monotherapy in BRCAmt patients or platinum-sensitive recurrence patients with homologous recombination deficiency (HRD). We present real-world experience from a single center of China.

**Methods:**

Patients treated with niraparib in Jiangsu Cancer Hospital between June 2019 to July 2020 were recruited. The initial dose was given according to individualization. Response and adverse events (AEs) were analyzed by Response Evaluation Criteria in Solid Tumors v1.1. and National Cancer Institute Common Terminology Criteria for Adverse Events v5.0, respectively. HRD testing (AmoyDx®) was detected in most patients. Treatment was given until unequivocal progression or intolerable toxicity.

**Results:**

Twenty-two patients all received niraparib at a bolus of 200 mg/d. Fifty percent of patients with high-grade serous ovarian cancer are HRD-positive. Six patients underwent first-line maintenance therapy. Sixteen patients received exploratory therapy. Ultimately image evaluation revealed that two patients achieved partial response (PR) and one patient achieved stable disease (SD), yielding objective response rate (ORR) of 33.3% (95%CI = 0.060–0.759) and disease control rate (DCR) of 50% (95%CI = 0.140–0.861) in the exploratory multi-line monotherapy group. The most common AEs were nausea, thrombocytopenia, and anemia. Grade 3–4 thrombocytopenia were managed by dose reduction and interruption. Leg swelling was observed as a new adverse event.

**Conclusion:**

It is feasible that patients receiving a bolus of 200 mg/d in patients from Chinese population can acquire promising efficacy and tolerance. This is the first real-world data about niraparib in ovarian cancer patients with available HRD status from China.

## Background

Ovarian cancer is the most lethal gynecological malignancy, 70% of which are diagnosed with advanced stage. Although most ovarian cancer patients are sensitive to standard first-line treatment including cytoreductive surgery and platinum-based chemotherapy, about 80% patients relapse within 1 to 2 years after initial treatment and gradually progress to platinum-resistance ovarian cancer, accompanied by significantly shortened survival [1, 2]. How to prolong the platinum free interval (PFI) becomes one of the breakthrough points in ovarian cancer treatment. Recently, poly ADP-ribose Polymerase (PARP) inhibitors have changed the treatment paradigm for ovarian cancer that can significantly improve the PFI, and finally prolonged the overall survival of patients with BRCA mutation [3–6].

PARP is a specific DNA fracture receptor, which is activated after DNA damage. PARP can recognize and bind to the DNA fracture site, mediating DNA single-strand damage repair in tumor cells [7]. PARP inhibitor can lead to DNA double strand damage inducing by amount of DNA single-strand damage. Normal cells can repair double-strand breaks through homologous recombination repair pathway. In tumor cells with homologous recombination deficiency (HRD), there are several genetic mutations such as BRCA mutation or other mutations in genes of homologous recombination repair (HRR) pathway (e.g., RAD51 and ATM). Due to treatment with PARP inhibitor, tumor cells can’t repair DNA single-strand damage and double-strand breaks, forming the synthetic lethal effect [8]. Therefore, BRCAmt or HRD-positive tumor cells are more sensitive to PARP inhibitors in terms of molecular mechanisms.

Niraparib (Zejula®) is a highly selective inhibitor of PARP1/2 (nuclear proteins that detect DNA damage and promote its repair) [9]. In 2017, it was firstly approved for second-line maintenance treatment of ovarian cancer patients who were in complete or partial response to platinum-based chemotherapy by Food and Drug Administration (FDA) according to the study of NOVA [3, 10]. Another study observed significant efficacy of niraparib in patients with newly diagnosed advanced ovarian cancer after response to first-line platinum-based chemotherapy [5]. Both of NOVA and PRIMA studies found that patients with HRD could get more benefits from niraparib. Recently the first fully powered, multi-center, phase III clinical study in Chinese population (NORA) showed that median PFS was significantly longer for niraparib as second-line maintenance treatment versus placebo among patients with germline BRCA mutations (not reached vs. 5.5 months; HR: 0.22) and those without germline BRCA mutations (11.1 vs. 3.9 months, HR: 0.40) in 2020 ESMO meeting [11]. QUADRA study demonstrated that niraparib brought great survival benefits among women with heavily pretreated ovarian cancer, especially in patients with HRD-positive platinum-sensitive disease, which included not only patients with BRCA mutation but also population with BRCA wild-type [12].

However, there was no real-world data to illustrate the efficacy and safety of niraparib in Chinese population. We conducted this study to assess the real-world experiences of niraparib in ovarian cancer patients with HRD status from our single center.

## Materials and methods

### Study population

Patients with ovarian cancer receiving niraparib from June 2019 to July 2020 in Jiangsu Cancer Hospital were included. We collected the baseline characteristics of these patients, including age, Eastern Cooperative Oncology Group performance status (ECOG PS) before the beginning of the treatment, histological type, clinical stage on the basis of International Federation of Gynecology and Obstetrics (FIGO), basal body weight, basal platelet count, previous therapy before and after niraparib treatment and the follow-up. The study was approved by the ethics committee of Jiangsu Cancer Hospital.

### Group standard

Patients who progressed during initial treatment, or completely/partially responded to initial treatment (cytoreductive surgery and platinum-based chemotherapy), but recurred within 6 months are defined as platinum-refractory/platinum-resistant ovarian cancer. Patients relapsed at more than 6 months after initial treatment are considered as platinum-sensitive ovarian cancer. According to the guidelines, patients treated with three or more prior lines of chemotherapy and whose cancer is associated with HRD positive defined by either: 1) a deleterious or suspected deleterious BRCA mutation; or 2) genomic instability and progression more than 6 months after response to the last platinum-based chemotherapy can use niraparib as multi-line therapy.

### Dosing regimen

The initial dose was based on the level of basal body weight and platelet count. Patients with basal body weight ≥ 77 kg and basal platelet count of ≥150,000/μl (μL) received 300 mg daily. While patients with basal body weight < 77 kg and/or basal platelet count< 150,000/μL received 200 mg daily. Dose reduction (300 mg to 200 mg or 100 mg; 200 mg to 100 mg) or interruption for drug-related AEs was allowed. Serum CA125 and imaging examinations were performed on each patient at baseline, followed by monthly examination of CA125 and bimonthly imaging examinations.

### HRD testing

The paraffin sections from the cytoreductive surgery were obtained after patients’ informed consent. DNA was extracted from FFPE biopsy/surgical specimens; 50 to 200 ng DNA undergoes library construction and hybrid capture with AmoyDx® HRD panel, which selected coding sequences (CDS) regions for 54 HRR pathway genes and 72,000 single nucleotide polymorphisms (SNPs) for HRD calling. The selected libraries were pooled and sequenced on the Illumina Novaseq6000 to > 500× unique coverage for 54 HRR genes and > 100× for SNP loci.

Sequence data was processed using a customized analysis pipeline designed to accurately detect multiple classes of genomic alterations: base substitutions, short insertions/deletions with detection sensitivity at variant allele frequency (VAF) ≥5%. Detected mutations were annotated according to American College of Medical Genetics (ACMG) guideline [13] and classified as pathogenic, likely pathogenic, variants of unknown significance, likely benign and benign. HRD score was calculated by the sum of three types of genomic instable events including loss of heterozygosity (LOH), telomeric allelic imbalance (TAI) and large-scale state transition (LST) defined by ref. [14]. HRD-positive was defined by either BRCA1/2 pathogenic or likely pathogenic mutation or HRD score ≥ 42.

### Assessments

Demographic and baseline data were summarized and analyzed. The efficacy was assessed as complete response (CR), partial response (PR), stable disease (SD) and progressive disease (PD) by RECIST 1.1. Objective response rate (ORR) was defined as the proportion of patients achieving CR or PR. Disease control rate (DCR) was defined as the proportion of patients achieving CR, PR or SD for at least 8 weeks. Treatment-related adverse events (AEs) were graded according to CTCAE 5.0.

### Statistical analysis

The 95% confidence interval was calculated using the Wilson procedure with a correction for continuity. Data were statistically analyzed using SPSS version 19.0 professional statistical software and all the count data were expressed as a percentage (%).

## Results

### Patients’ characteristics

A total of 22 patients treated with niraparib were enrolled, included 21 patients with ovarian cancer and 1 patient with fallopian tube cancer. The median age was 55.0 years (range 39–77 years). Patient demographics and baseline characteristics were listed in Table 1. FIGO stage II, III and IV, affected 2 (9.1%), 11 (50.0%) and 8 (36.4%) of patients, respectively. Most patients (86.4%) were high-grade serous cancer. All participants weighed less than 77 kg, 10 of who had basal platelet count less than 150,000 per cubic millimeter. The results of HRD testing were positive in six patients and negative in eight patients.

**Table 1:** Baseline characteristics in 22 patients. Values are reported as frequency (n [%]) or as mean (range)

Characteristic	Number of patients (percent)
Age, yrs	
Median age (range)	55 (39–77)
≤ 55	12 (54.5)
> 55	10 (45.5)
Primary tumor location	
Ovary	21 (95.5)
Fallopian tube	1 (4.5)
International FIGO stage	
II	2 (9.1)
III	11 (50.0)
IV	8 (36.4)
Unknown	1 (4.5)
Histological type	
High-grade serous	19 (86.4)
Low-grade serous	1 (4.5)
Other	1 (4.5)
Unknown	1 (4.5)
Family history of cancer	
Yes	9 (40.9)
No	13 (59.1)
ECOG	
0	8 (36.4)
1	13 (59.1)
2	1 (4.5)
Baseline body weight	
≥ 77 kg	0 (0)
< 77 kg	22 (100)
Platelet count	
≥ 150 × 10^9^/L	12 (54.5)
< 150 × 10^9^/L	10 (45.5)
HRD status	
HRD-positive	6 (27.3)
BRCA-mutated	1 (4.5)
BRCA-wild type or BRCA-unknown and HRD-positive	5 (22.7)
HRD-negative	8 (36.4)
HRD unknown	8 (36.4)
Prior lines of chemotherapy	
≤ 1	12 (54.5)
> 1	10 (45.5)
Platinum status	
Platinum-sensitive	5 (22.7)
Platinum-resistant	5 (22.7)
Unknown	12 (54.5)
Categories of therapy	
First-line maintenance therapy	6 (27.3)
Exploratory therapy	16 (72.7)
Exploratory second-line maintenance therapy	1 (4.5)
Exploratory front-line therapy	6 (27.3)
Exploratory multi-line therapy	9 (40.9)
NACT+IDS	
Yes	7 (31.8)
No	15 (68.2)
Primary debulking surgery	
Yes	12 (54.5)
No	10 (45.5)
Secondary cytoreductive surgery	
Yes	3 (13.6)
No	19 (86.4)
Combination with other agents	
Yes	4 (18.2)
No	18 (81.8)

*Abbreviations*: *FIGO* International Federation of Gynecology and Obstetrics, *ECOG* Eastern Cooperative Oncology Group, *HRD* homologous recombination deficiency, *NACT* Neoadjuvant chemotherapy, *IDS* Interval debulking surgery

### Group assignment

On the basis of group standard, the patients were divided into first-line maintenance treatment group and exploratory treatment group (not in the scope of indications), with 6 and 16 patients in each group respectively. For exploratory therapy, there were three subgroups including exploratory second-line maintenance, front-line (less than three chemotherapy regimens) and multi-line (three or more prior chemotherapy regimens) treatment group, with one, six and nine patients in each group, respectively.

### Efficacy, CA125 and HRD status

**Fig. 1:**
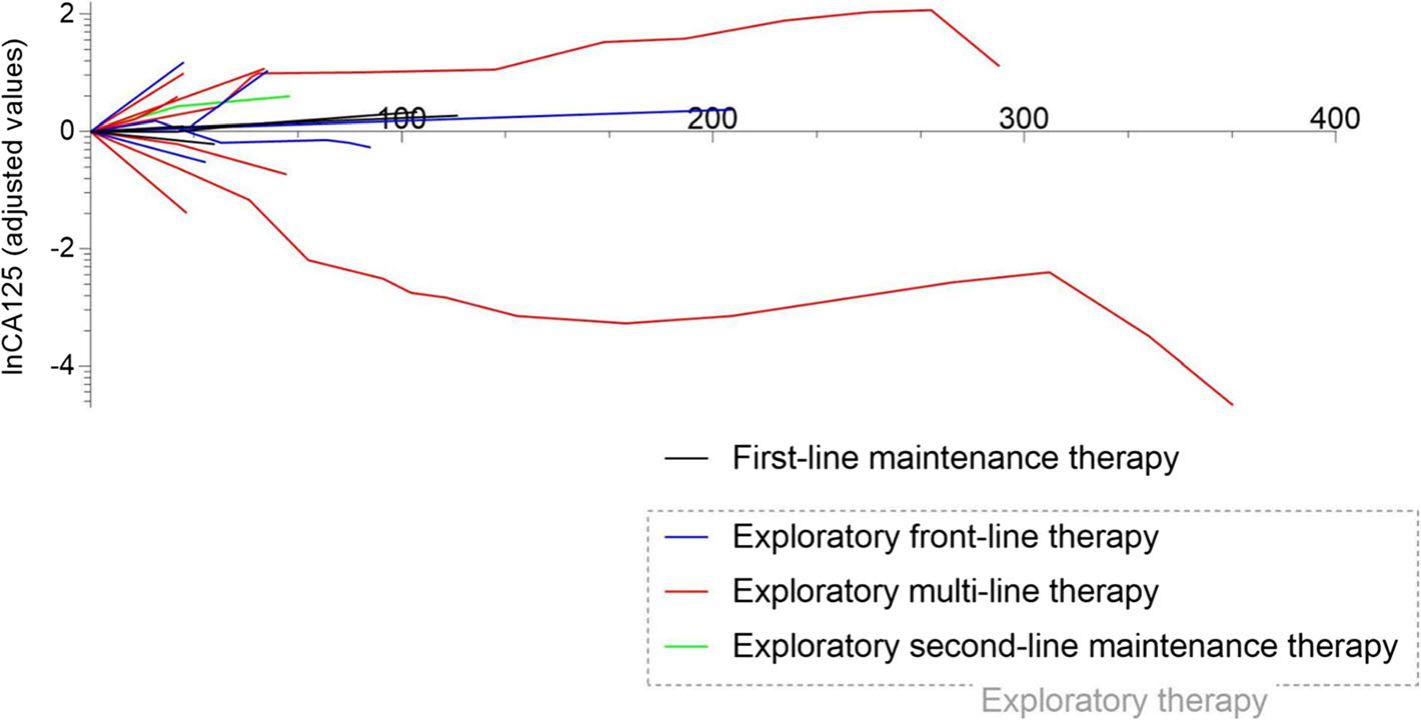
Serum CA125 values in each group. Note: The CA125 level of the first follow-up was used as the reference value, and all data were converted to natural logarithm. CA125 follow-up data were obtained from 6 patient in the first-line maintenance treatment group and 16 patients in the exploratory therapy group

In first-line maintenance treatment group, all six patients are still on medication, the median follow-up time of whom is 18 weeks (range 8–44 weeks). Among these, two cases were HRD-positive while HRD test was not conducted for the rest four patients. In the exploratory treatment group, one HRD-negative patient diagnosed with highly differentiated papillary mesothelioma has been receiving niraparib as the second-line maintenance treatment. Two cases in exploratory front-line therapy and 9 cases in exploratory multi-line therapy were also tested for HRD. The remaining patients were not underwent HRD tests. Serum CA125 of these patients with maintenance treatment were shown in Fig. 1.

Among the different therapeutic strategies in the exploratory first-line therapy subgroup, three patients who did not undergo surgery and only received chemotherapy for their personal willingness achieved SD after treated with niraparib, of these 2 patients with HRD positive and HRD negative, respectively. All three patients are still assessed as SD. Two patients with first platinum-sensitive recurrence ovarian cancer achieved SD, one of which was treated with niraparib monotherapy and the other one was treated with niraparib and anlotinib. One patient who did not receive chemotherapy after the surgery due to poor renal function achieved PD.

**Fig. 2:**
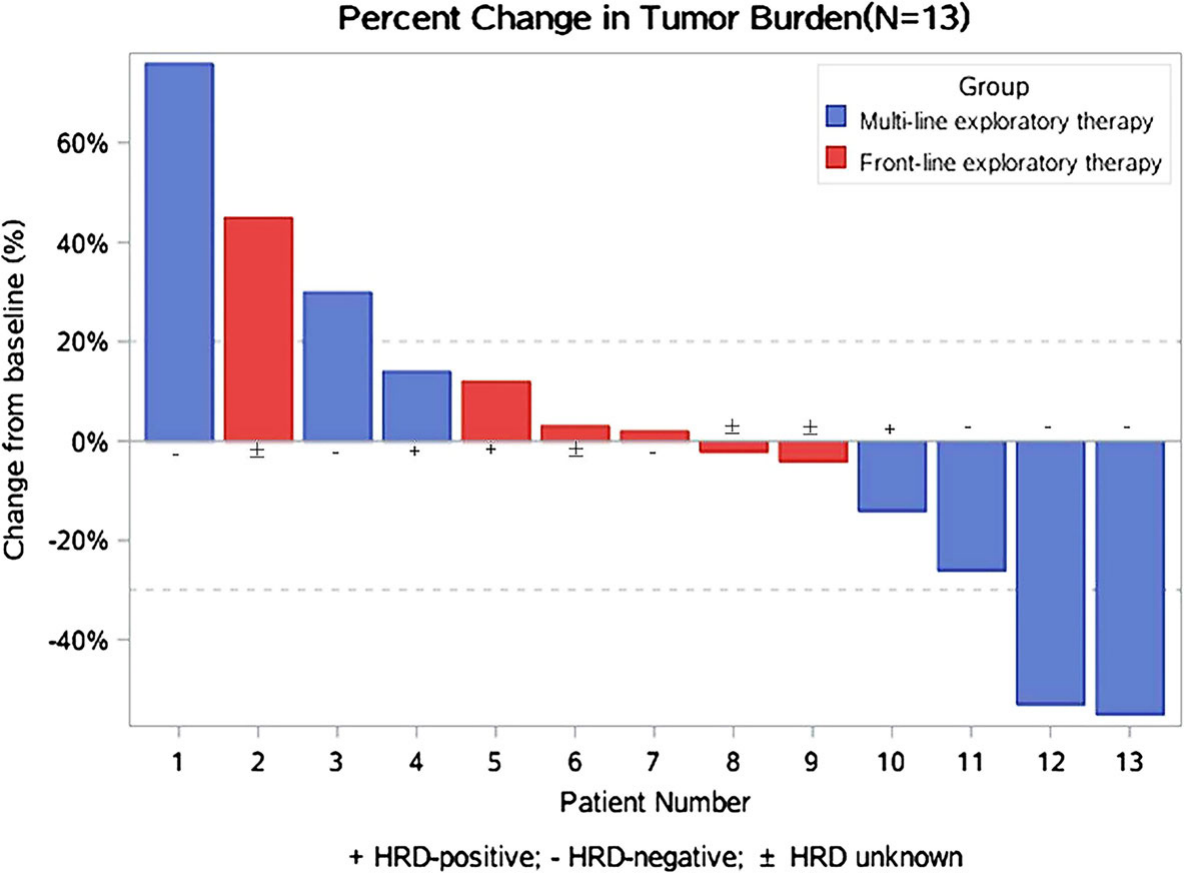
Waterfall plot of 13 evaluable patients with exploratory therapy. Note: Tumor burden change per investigator review. Maximum reduction from baseline (or smallest increase from baseline for patients with no reductions) in the sum of the longest diameters of target lesions. The change from baseline in tumor measurement as assessed by investigator review is shown for 13 evaluable patients (per protocol set). Front-line exploratory subgroup and multi-line exploratory subgroup were shown with different color in the figure. Among them, there were 3 HRD-positive patients, 6 HRD-negative patients and 4 HRD-unknown patients. Ten of them were treated by niraparib monotherapy while 3 of them (patient 1,7,11) were treated by combined strategies. The dotted line represents the threshold for partial response (> 30% reduction from baseline sum of longest diameters) and progressive disease (> 20% increase from baseline sum of longest diameters). Target lesions were defined according to RECIST 1.1

The median prior line was 5 (range 3–8) in the exploratory multi-line therapy subgroup. Ultimately therapeutic evaluation showed that two patients achieved partial response (PR), one patient achieved stable disease (SD) and three patients had progressive disease (PD), yielding the objective response rate (ORR) of 33.3% (95%CI = 0.060–0.759) and the disease control rate (DCR) of 50% (95%CI = 0.140–0.861) in patients with exploratory multi-line monotherapy. There were also three patients treated with exploratory multi-line combination therapy, one patient of whom achieved SD using niraparib combined with topotecan and anlotinib but another one failed with this combination therapy. The remaining patient achieved PD by niraparib combined with anlotinib (Table 2). The available HRD status and tumor shrinkage in the exploratory front-line and multi-line treatment subgroups were listed in Fig. 2.

**Table 2:** Short-term efficacy of 9 evaluable patients with exploratory multi-line therapy

Short-term efficacy	Monotherapy, n (%)	Combined Treatment, n (%)
Complete response (CR)	0 (0)	0 (0)
Partial response (PR)	2 (33.3)	0 (0)
Stable disease (SD)	1 (16.7)	1 (33.3)
Progression disease (PD)	3 (50.0)	2 (66.7)
Objective response rate (ORR)	2/6 (33.3)	0 (0)
Disease control rate (DCR)	3/6 (50.0)	1/3 (33.3)

The table above showed the short-term efficacy of 9 evaluable patients with exploratory multi-line therapy including 6 patients with niraparib monotherapy and 3 patients with combined treatment. Short-term efficacy was classified by modified Response Evaluation Criteria in Solid Tumors version 1.1 (RECIST 1.1)

### Safety

The most common AEs were nausea (55.6%), thrombocytopenia (44.4%), anemia (33.3%), and decreased appetite (33.3%), while serious AEs (SAEs) were thrombocytopenia (16.7%), anemia (5.6%), neutropenia (5.6%), vomiting (5.6%) and dyspepsia (5.6%) in patients with niraparib monotherapy. Three patients with thrombocytopenia, neutropenia, or vomiting relieved through dose interruption followed by dose reduction (200 mg to 100 mg). One patient with dyspepsia could not tolerate drug-related AE after dose reduction and discontinued the treatment. In patients with niraparib combined therapy, the most common AEs were thrombocytopenia (75.0%), anemia (75.0%), fatigue or asthenia (75.0%), nausea (50.0%) and neutropenia (50.0%), while SAEs were thrombocytopenia (25.0%), anemia (25.0%) and neutropenia (25.0%). One patient received the combination therapy including niraparib, topotecan and anlotinib suffered grade 4 thrombocytopenia within 1 week. She stopped taking the medicine and were treated with niraparib monotherapy at a dose of 200 mg daily after elevation of platelet count. Another patient treated with niraparib in combination with topotecan and anlotinib also suffered grade3–4 anemia and had a dose reduction and interruption. The remaining two patients treated with niraparib plus anlotinib suffered grade1–2 AEs. SAEs were not observed in two patients with initial dose of 100 mg daily. We observed leg swelling as a new adverse event in one patient. Summary of AEs including niraparib monotherapy and niraparib combined therapy were listed in Table 3.

**Table 3:** Summary of adverse events

Adverse event	Niraparib monotherapy (*n* = 18)		Niraparib combined therapy (*n* = 4)	
	Any grade	Grade 3 or 4	Any grade	Grade 3 or 4
	Number of patients (percent)			
Nausea	10 (55.6)	0 (0)	2 (50.0)	0 (0)
Thrombocytopenia	8 (44.4)	3 (16.7)	3 (75.0)	1 (25.0)
Anemia	6 (33.3)	1 (5.6)	3 (75.0)	1 (25.0)
Decreased appetite	6 (33.3)	0 (0)	1 (25.0)	0 (0)
Fatigue or asthenia	5 (27.8)	0 (0)	3 (75.0)	0 (0)
Constipation	5 (27.8)	0 (0)	1 (25.0)	0 (0)
Insomnia	5 (27.8)	0 (0)	0 (0)	0 (0)
Neutropenia	3 (16.7)	1 (5.6)	2 (50.0)	1 (25.0)
Vomiting	3 (16.7)	1 (5.6)	0 (0)	0 (0)
Dyspepsia	3 (16.7)	1 (5.6)	0 (0)	0 (0)
Headache	2 (11.1)	0 (0)	1 (25.0)	0 (0)
Abdominal distention	2 (11.1)	0 (0)	0 (0)	0 (0)
Dizziness	1 (5.6)	0 (0)	1 (25.0)	0 (0)
Dysgeusia	1 (5.6)	0 (0)	0 (0)	0 (0)
Back pain	1 (5.6)	0 (0)	0 (0)	0 (0)
Diarrhea	1 (5.6)	0 (0)	0 (0)	0 (0)
Maculopapular rash	1 (5.6)	0 (0)	0 (0)	0 (0)
Stomatitis	1 (5.6)	0 (0)	0 (0)	0 (0)
Dry mouth	1 (5.6)	0 (0)	0 (0)	0 (0)
Abdominal pain	0 (0)	0 (0)	1 (25.0)	0 (0)
Newly observed				
Leg swelling	1 (5.6)	0 (0)	0 (0)	0 (0)
Led to discontinuation of intervention	1 (5.6)	–	0 (0)	–
Led to dose reduction	3 (16.7)	–	1 (25.0)	–
Led to dose interruption	2 (11.1)	–	2 (50.0)	–

Note: Adverse events were graded according to National Cancer Institute Common Terminology Criteria for Adverse Events (NCI CTCAE), version 5.0

## Discussion

PARP inhibitor is a major advance in the treatment of ovarian cancer. Patients with BRCA mutation or HRD positive can get more benefit from it. Now there are two kinds of PARP inhibitors including olaparib and niraparib in China. We previously reported the first real-word study of olaparib in Chinese population [15]. Here we presented the first real-word experience of niraparib for ovarian cancer patients from China.

A retrospective analysis of ENGOT-OV16/NOVA trial suggested that patients with baseline body weight < 77 kg or baseline platelets < 150,000/ml might benefit from a starting dose of 200 mg daily [16]. A subsequent study proved that incidence of common reported AEs of clinical trials were lower among patients initiating niraparib 200 mg/d in real-world practice versus patients initiating niraparib 300 mg/d in Caucasian population from ENGOT-OV16/NOVA study [17]. Recently the NORA trial demonstrated that niraparib maintenance therapy administered with an individual starting dose regimen, most subjects received the initial dose of 200 mg/d, significantly improved the outcome in patients with recurrent ovarian cancer in 2020 ESMO meeting [11]. All patients in our real-world experience receiving 200 mg/d according to the basal weight and basal platelet count was consistent with the results of prospective studies in Chinese population.

At the 2020 ASCO meeting, an open-label, non-randomized study (LIGHT) showed that patients with platinum-sensitive recurrence, high-grade serous/endometrioid epithelial ovarian cancer and one or more prior lines of platinum chemotherapy could benefit from olaparib monotherapy, especially in patients with HRD-positive [18]. Similarly, in our exploratory front-line therapy subgroup, two patients with first platinum-sensitive recurrence achieved SD, one of which was with HRD-positive and the other was with HRD-negative. This finding needs to be confirmed by a prospective study of niraparib as front-line monotherapy.

The PAOLA-1 trial suggested that olaparib combined with bevacizumab provided a significant progression-free survival benefit to advanced ovarian cancer patients receiving first-line standard therapy including bevacizumab, which was substantial in patients with HRD positive, including those without BRCA mutation [19]. Niraparib plus bevacizumab significantly improved progression-free survival compared with niraparib alone in platinum-sensitive recurrent ovarian cancer [20]. One HRD-negative patient also achieved SD receiving niraparib plus anlotinib which might be related to the synergistic antitumor effect of PARP inhibitors and antiangiogenic drugs. Further studies are also needed to observe efficacy of the combination strategies in patient with HRD negative, regardless of front-line therapy or multi-line therapy.

A multi-center, open-label, single-arm, phase 2 QUADRA trial observed that 10 (27%) of 37 platinum-resistant patients harbored BRCA mutation, 12 (10%) of 120 platinum-resistant patients with HRD positive and 5 (3%) of 169 platinum-resistant patients with HRD negative achieved an overall response according to RECIST1.1 in the primary analysis of efficacy [12]. Regardless of the patients’ BRCA status or HRD status, our results showed the ORR of 33.3% (95%CI = 0.060–0.759) and the DCR of 50% (95%CI = 0.140–0.861) respectively in platinum-resistant ovarian cancer patients with exploratory multi-line monotherapy. We consider the differences of response to niraparib may be due to the small number of patients enrolled in our study and the criteria for enrollment in our real-world data.

Previous studies confirmed that ovarian cancer patients with HRD positive were more likely to benefit from niraparib than those with HRD negative. We didn’t observe the obvious relationship between efficacy and HRD status among our available cases, which may be correlated with our small sample size. One study reported that approximately 50% of patients with high-grade serous ovarian cancer are HRD-positive [21]. Among all enrolled cases in our study, 21 participants of whom were BRCAwt or BRCA unknown, and 14 cases were tested with HRD panel. Of the 12 patients with high-grade serous ovarian cancer, 6 patients were with HRD- positive and 6 patients were HRD-negative. The results of our report were consistent with the study of large sample.

The most common AEs were nausea, thrombocytopenia, anemia and fatigue. SAEs were thrombocytopenia, anemia, neutropenia and dyspepsia for both niraparib monotherapy and combined therapy that was higher in latter. The incidence of AEs and SAEs in our observation were similar to other studies. A meta-analysis of current clinical trials also showed the same characteristics of AEs [22]. All SAEs occurred within 1 month after receiving therapy with niraparib, most of which occurred within 1 week. The most common AEs caused dose reduction and interruption of treatment were serious thrombocytopenia and vomiting. We also observed that one patient suffered grade 1 leg swelling in the second week after treatment with niraparib 200 mg daily, the mechanism of which need to be explored in future.

Severe myelosuppression including thrombocytopenia and neutropenia, and vomiting were alleviated by dose reduction and interruption. Only one patient with dyspepsia could not tolerate after dose reduction to 100 mg/d and discontinued the treatment. With respect to PARP inhibitors combined with chemotherapy, the GOLD study did not meet its primary objective of showing a significant improvement in overall survival with olaparib in combination with a chemotherapeutic agent and in the overall or ATM-negative population of Asian patients with advanced gastric cancer due to the intolerable AEs [23]. In our study, one patient was given the combination therapy including niraparib, topotecan and anlotinib. She suffered grade 4 thrombocytopenia within 1 week. Similar to the GOLD study, we also observed the intolerable AEs in patients using PARP inhibitors combined with chemotherapy.

## Conclusion

This is the first real word data about niraparib in ovarian cancer patients with HRD status from China. Our findings demonstrated that Chinese population with niraparib 200 mg orally once daily is feasible. Leg swelling was observed as a new adverse event in our study. HRD tests in our small samples confirmed that 50% of patients with high-grade serous ovarian cancer were HRD-positive. However, our data are limited representative due to limited number of cases. Further clinical trials are needed to verify the exploratory therapy in our study.

## Data Availability

We would not share the data and material used in this manuscript, because we need them for further research.

## Abbreviations

PARP: Poly (ADP-ribose) polymerase
HRD: Homologous recombination deficiency
HRR: Homologous recombination repair
PFI: Platinum-free-interval
RECIST: Response Evaluation Criteria in Solid Tumors
